# Variations of the metabolome in the digestive system of Antarctic krill, *Euphausia superba*, between summer and autumn

**DOI:** 10.1371/journal.pone.0327747

**Published:** 2025-07-10

**Authors:** Simone Heyen, Verena Töpker, Bernd Kopke, Nevenka Cakić, Lukas Hüppe, Bettina Meyer, Heinz Wilkes

**Affiliations:** 1 Institute for Chemistry and Biology of the Marine Environment (ICBM), Carl von Ossietzky University of Oldenburg, Oldenburg, Germany; 2 Alfred Wegener Institute for Polar and Marine Research, Bremerhaven, Germany; 3 Julius-Maximilians-University of Würzburg, Würzburg, Germany; 4 Helmholtz Institute for Marine Functional Biodiversity (HIFMB), Carl von Ossietzky University of Oldenburg, Oldenburg, Germany; Karlsruhe Institute of Technology: Karlsruher Institut fur Technologie, GERMANY

## Abstract

Rapid climate change threatens the relatively pristine environment of the Southern Ocean. The effects on biogeochemical cycles and their subsequent consequences for the organisms in this area are of significant interest. Antarctic krill, *Euphausia superba*, is the dominant species in the Antarctic ecosystem and a vital component of the Southern Ocean food web. Its metabolism is influenced by the feeding regime, which is governed by environmental conditions. However, little is yet known about the metabolome of Antarctic krill. Here, we investigated metabolite classes that can serve as tracers for documenting variations in the metabolic and biochemical status of krill. To this end, we utilised targeted metabolomics to analyse coenzyme A thioesters, amino acids, B vitamins, and respiratory quinones in the digestive system of Antarctic krill, sampled during two campaigns in the Antarctic summer and autumn. A significant proportion of the detected coenzyme A thioesters were associated with the β-oxidation of fatty acids and, consequently, with lipid metabolism. Propionyl-CoA was particularly abundant in samples from the digestive gland, while malonyl- and succinyl-CoA were more prevalent in stomach and hindgut samples. 3-Hydroxy-3-methylglutaryl-CoA, an intermediate in the metabolism of branched amino acids and the biosynthesis of isoprenoids, occurred almost exclusively in the summer samples. Analyzing the free amino acids, very high levels of the non-proteinogenic amino acid sarcosine were found, which possibly serves as an osmolyte for the Antarctic krill and/or plays a role in its digestive process. Among the B vitamins, there were seasonal fluctuations, particularly in B_1_ and B_5_. The respiratory quinones exhibited more homogeneous patterns, with UQ_10:10_ as the dominant representative. These seasonal and organ-dependent variations in the composition of the different metabolite classes can serve as a reference point in future studies to better assess the influence of changing conditions in Antarctic waters.

## Introduction

Antarctic krill (*Euphausia superba,* hereafter referred to as krill) is a small, shrimp-like crustacean that plays a crucial role in the ecosystem of the Southern Ocean, serving as an essential part of the diet for many marine animals and occupying an important role in the biogeochemical cycles of Antarctic waters [1]. Krill is a highly abundant species in the Southern Ocean, with an enormous biomass of approximately 379 million tonnes [2]. Its nutritional value, derived from characteristics such as a high content of essential omega-3 fatty acids, makes it appealing for medical applications and highlights its importance as a subject of study [3,4].

Krill are filter feeders, consuming food particles that are filtered through the feeding basket formed by their thoracopods [5]. After being initially crushed in the mouthparts, food items are further ground in the gastric mill of the stomach, filtered, and passed into the digestive gland [6,7]. Enzymes break down the filtered chyme in the digestive gland before the remains are compacted into fecal pellets and excreted through the hindgut. The initial breakdown of complex materials in the stomach, such as chitinous food, is indicated by elevated endochitinase activity, with subsequent degradation to amino sugars occurring in the digestive gland [8]. It has also been shown that the activity of digestive enzymes is generally higher in the stomach than in the digestive gland [8–10].

Due to changes in food availability, the efficiency of food absorption can vary greatly between seasons. This is reflected in changes in egestion rates, densities, and C:N ratios in fecal pellets [11]. Conversely, the composition of metabolic products in the digestive system will likely also depend on the food ingested by krill across seasons. These seasonal variations have already been documented in the compositions of lipids and fatty acids, suggesting that seasonal patterns may also occur in other metabolite classes [12–15].

Metabolites play vital roles in biological processes, including energy production, growth, reproduction, and responses to environmental stimuli. Consequently, the metabolome of krill can reflect the diverse biochemical processes occurring within these organisms. It can vary based on several factors, such as diet, environmental conditions, and life stage.

As ongoing climate change affects the habitat and food availability of krill, understanding their metabolome is of great value in studying their biology and ecological roles [16,17]. While considerable research has been conducted on krill’s lipid and fatty acid composition, limited data exist on other classes of metabolites [13,15,18,19]. The first untargeted metabolomic study was conducted by Zhang et al., which focused on contaminants found in krill tissues [20]. Thus, there remains significant potential to expand knowledge of the krill’s metabolome, which prompted us to analyse key metabolite classes that may exhibit seasonal patterns due to changes in food supply. These included coenzyme A thioesters (acyl-CoAs), amino acids, B vitamins, and respiratory quinones.

Acyl-CoAs are essential intermediates involved in numerous metabolic pathways within cells. They function as carriers of acyl groups in metabolic reactions, playing a vital role in energy metabolism, lipid metabolism, and the synthesis and degradation of various cellular constituents. Amino acids are crucial for protein synthesis and play a role in metabolism, immune response, and other physiological processes. They serve as precursors for synthesising various bioactive compounds and are involved in signalling pathways and enzymatic reactions. The organism cannot synthesize essential amino acids, which must be obtained from the diet, while non-essential amino acids can be synthesized within the body. B vitamins are necessary for energy metabolism, the nervous system’s function, and DNA synthesis, among other functions. Respiratory quinones shuttle electrons between different respiratory chain complexes and thus play a role in ATP synthesis. While there are no comprehensive studies on the composition of acyl-CoAs, B vitamins, and respiratory quinones in krill, studies on the amino acid content and composition have mainly focused on their nutritional value [21–23].

## Materials and methods

### Krill samples

Krill were sampled during two expeditions (03.05.-17.06.2021 and 02.01.-26.03.2022) aboard the commercial krill fishing vessel (FV) *Antarctic Endurance* of the company Aker BioMarine. This beam trawler is equipped with a continuous fishing system that uses a vacuum system to pump krill from the cod end of two nets on board. The first collection took place on the 13^th^, 15^th^ and 31^st^ of May (autumn) of 2021 in the Bransfield Strait, while the second occurred on the 19^th^ of January and 8^th^ of March (summer) of 2022 at the South Orkney Islands. Upon capture, the stomach, digestive gland, and hindgut of krill individuals were immediately dissected on board under a stereomicroscope, frozen, and stored at −80°C for later analysis. Individual krill specimens were utilized for the assessment of amino acids. At the same time, body parts collected and pooled from three animals were used for the analysis of acyl-CoAs, B vitamins, and respiratory quinones. Five to twelve replicates were used for each sampling time point and organ.

### Extraction

#### Bead beating.

To avoid autolytic processes and thus alterations of the metabolite composition, sample transportation and extraction was performed under dark conditions using minimal artificial light as well as storage on dry ice when samples were not actively treated [24]. Bead-beating was used for the extraction of all samples. The respective internal standards as listed in Table 1 were added prior to the individual extraction procedures.

**Table 1:** Internal standards used for individual metabolite classes.

Metabolite class	Internal standard
Coenzyme A thioesters	Benzoyl-CoA-^13^C_6_^a^
B vitamins	Pantothenic acid-^13^C_3_,^15^N^b^
Amino acids	Leucine-d_10_
Respiratory quinones	1,2-Distearoyl-d_70_-sn-glycero-3-phosphocholine

^a^ Benzoyl-CoA-^13^C_6_ was synthesized and purified from benzoic acid-^13^C_6_ as described in Cakić et al. [25].

^b^ The β-alanine moiety of pantothenic acid was ^13^C-labelled.

For samples intended for analysis of respiratory quinones, a mixture of methanol (MeOH) and hexane (2:1) was used, while all other samples were extracted with MeOH. Tissue samples were transferred into cryotubes filled with 0.5 g of 0.1-mm glass beads and 0.7 g of 0.7-mm zirconia beads. Bead-beating was performed with 1 mL of solvent in three cycles of 30 seconds with intervening cooling phases on dry ice of 90 seconds. After centrifugation and removal of the solvent, two additional rounds of extraction were done by re-adding 0.5 mL solvent, vortexing, and centrifuging the sample again. The entire procedure was repeated twice, resulting in a total extract volume of 6 mL. Depending on the targeted metabolite class, the subsequent extraction process was adjusted accordingly.

#### Coenzyme A thioesters and B vitamins.

For acyl-CoAs and B vitamins, bead-beater MeOH extracts were evaporated to dryness under a nitrogen stream, resuspended in ultrapure water, and filtered using centrifuge filters. The filtrates were stored at −80°C.

#### Amino acids.

Hydrolysis of protein-bound and derivatization of protein-bound as well as free amino acids was conducted according to Vane et al. and Walsh et al. [26,27]. For this procedure, bead-beater MeOH extracts were evaporated to dryness and resuspended in 1 mL MeOH. 100 µL each for the hydrolysis as well as the direct derivatization were placed in conical vials with a capacity of 500 µL and evaporated. In case of the hydrolysis of protein-bound amino acids, the samples were resuspended in 200 µL of 6 M hydrochloric acid (HCl). To prevent oxidation, 40 µL of ascorbic acid were added before flushing the vials with nitrogen and placing them in the oven at 110°C for 20 h. After cooling, the samples were evaporated to dryness. The following derivatization procedure for these samples as well as for those not hydrolysed was similar. The samples were resuspended in 200 µL of 0.1 M HCl before 70 µL of MeOH, 60 µL of pyridine and 30 µL of methyl chloroformate were added. Subsequently, 200 µL of chloroform were added to each sample, the sample was mixed and the lower layer containing methoxycarbonyl (MOC) esters was dried over sodium sulphate after waiting for phase separation.

#### Respiratory quinones.

Respiratory quinones were extracted based on the method described by Tindall [28]. Bead-beater extracts were stirred for 30 minutes at room temperature in the dark. Ice-cold hexane was added to change the ratio of MeOH and hexane to 1:1 (v:v) and the sample was vortexed. After incubating on ice for 15 minutes, the samples were centrifuged, and the hexane phase was transferred to a glass vial. 2 mL of hexane and 2 mL of aqueous 0.3% sodium chloride were added to the samples and mixed. Succeeding another round of centrifugation, the hexane phases were pooled and evaporated under a stream of nitrogen. The samples were resuspended in hexane:MeOH (1:10 v:v), filtered, and stored at −80°C until measurement.

### Instrumental analyses

#### Gas chromatography-mass spectrometry.

Gas chromatographic-mass spectrometric analysis of the derivatized amino acids was executed based on the method described in Heyen et al. on a Trace GC Ultra gas chromatograph coupled to a TriPlus autosampler and an ISQ QD mass spectrometer (all from Thermo Scientific, Dreieich, Germany) with an optimized temperature gradient [29]. The temperature program started at a temperature of 60°C, which was held for 1 min and then increased first at a rate of 3 K/min to 250°C and then at a rate of 10 K/min to 320°C, which was held for further 15 min.

#### Liquid chromatography – mass spectrometry.

B vitamins were measured on an Ultimate 3000 HPLC coupled to a TSQ Quantum UltraAM mass spectrometer (both Thermo Fisher Scientific, Dreieich, Germany). Separation was achieved on a Kinetex Evo C_18_ column (150 x 2 mm, 2.6 µm, Phenomenex, Torrance, CA, USA). Settings for the chromatography and the mass spectrometric method operated in selected reaction monitoring mode were adopted from Bruns et al. [30].

Acyl-CoAs and respiratory quinones were analysed using a Vanquish Flex UHPLC connected to an Orbitrap Fusion mass spectrometer (both Thermo Fisher Scientific, Dreieich, Germany). Acyl-CoAs were measured and identified as described in Cakić et al. on a Gemini C18 column (150 × 2.0 mm, 3 μm pore size, Phenomenex, Torrance, CA, USA) [25]. Reference standards were either purchased from a supplier or synthesized as described in Cakić et al. [25].

For the separation of respiratory quinones, the same column was used as for the B vitamins, with a chromatographic method based on Zhu et al. [31]. The eluents consisted of MeOH:H_2_O (85:15 v:v) (eluent A) and isopropanol (eluent B), both with a 10 mM ammonium acetate puffer at pH 8.1. Gradient started at 100% A for 2.5 min, which was increased to 15% B by 2.64 min. After increasing to 85% B in 22.49 min and to 100% B in 1.38 min, which was held for 6.25 min, the gradient went back to 100% A in 2.25 min, where it was held for 7.6 min. The total runtime was 42.6 min at a flow rate of 0.3 mL/min. For mass spectrometric analysis, initial measurements were conducted to assess the qualitative composition of respiratory quinones in krill samples based on characteristic fragmentation patterns as described by Elling et al. [32]. Samples with up to 15 pooled organs were used to assemble a targeted method in order to enhance the sensitivity of the measurements. These targeted MS^2^ measurements were run in parallel to full scan experiments for all samples. Instrument settings for both modes are summarized in S1 and S2 Tables in the supporting information.

### Data analysis

Peaks were integrated using the Xcalibur Quan Browser or TraceFinder (for acyl-CoAs), version 4.1.31.4 (Thermo Scientific, Dreieich, Germany). For acyl-CoAs and respiratory quinones, the internal standard was used to obtain semi-quantitative data, as no authentic standards were available, so that response and linearity differences within the targeted compound class were not taken into account. For the B vitamins and amino acids, a corresponding authentic standard was used for the calibration for each analyte. Amino acids were calibrated using 15 standards with concentrations between 0.01 and 30 µg/mL with and without hydrolysis. Since the tyrosine standard precipitated during determination of calibration curves due to poor solubility, the inclusion of this amino acid would have led to an overestimation of the content and it was therefore excluded. Methionine and cysteine were destroyed during hydrolysis and are excluded from the evaluation of the composition of the protein-bound fraction. Asparagine and any glutamine present were transformed to their corresponding acids during hydrolysis. Concentrations of ornithine, tryptophan, and lysine were below the quantification limit and excluded from further analysis.

For B vitamins, individual calibration ranges were selected based on the expected concentrations in the samples. These ranges as well as the number of levels are listed in S3 Table. Calibrations for both B vitamins and amino acids were generated in quadruplicate, with outliers eliminated applying the Grubbs test at a significance threshold of 0.05. For each area ratio of a B vitamin or amino acid to the internal standard in a sample, five calibration levels were selected to calculate the concentration. Process blanks were subtracted from all samples. B_3_ was detected in some samples, but due to high matrix loading, especially in the summer samples, peaks were difficult to integrate correctly. To avoid over- or underestimation of the content, B_3_ was removed from the evaluation.

Since it was not possible to weigh the samples without causing significant changes in the composition emerging from autolytic processes and considering that the weights of body parts are likely to vary by month, sex, and krill size, the evaluations were conducted solely based on the percentage distributions of the analytes in each sample. Although the samples were segregated based on sex, no discernible patterns were observed, so this variable was not considered in the subsequent analysis.

Statistical analyses were performed using the non-parametric Kruskal-Wallis and Dunn’s tests. Calculations and principal component analyses (PCA) for every class of metabolite were performed in R version 4.2.2, with the package ggbiplot used for visualisation [33,34]. PCA was used to identify trends and patterns in the data sets in addition to bar graphs, as the distribution of the data in the PCA plots is driven by metabolites with dominant influences on the sample. The percetanges given on each axis represent the proportion of variance explainable by the component. Results of the statistical tests as well as PCA plots can be found in the supporting information.

## Results and discussion

### Coenzyme A thioesters

**Fig 1:**
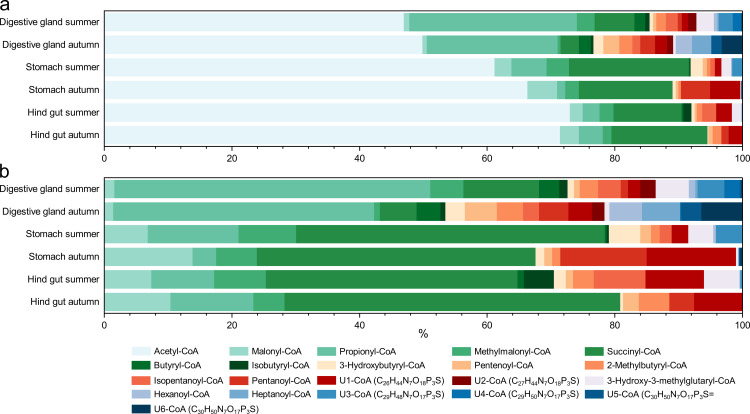
Relative distribution of coenzyme A thioesters with an abundance above 1% between organs and seasons. **a.** Including acetyl-CoA and **b.** excluding acetyl-CoA. Acyl-CoAs without confirmation of the structure by comparison with a reference standard are numbered as U1- to U6-CoA, with their sum formulas in parentheses.

To our knowledge, acyl-CoAs have not been studied in krill or other crustacean species. Therefore, this is the first time such a dataset can be presented. We detected 97 acyl-CoAs, ranging from acetyl-CoA to the CoA ester of a C_12_ acid that contains an additional oxygen atom. Thirty-one of these acyl-CoAs were identified by comparison with reference standards that were either commercially available or synthesised in-house. The remaining acyl-CoAs were characterised according to their sum formulas derived from high-resolution mass spectrometry. A list of all peaks, their respective sum formulas, and acyl-rests is provided in S4 Table. Their relative distribution is displayed in S1 Fig The distribution of 21 acyl-CoAs, which had a relative abundance of more than 1% among all 97 detected acyl-CoAs in at least one organ, is shown in Fig 1.

In all samples, acetyl-CoA constituted over 40% of the total acyl-CoA content (Fig 1a). Given that acetyl-CoA is a vital intermediate in numerous metabolic pathways, essential for both energy conservation and the biosynthesis of cellular constituents, this was to be expected. However, the proportion of acetyl-CoA increases from the digestive gland via the stomach to the hind gut. To better illustrate the compositional variability of the other acyl-CoAs, Fig 1b presents their distribution, excluding acetyl-CoA.

The most diverse pattern was observed in samples taken from the digestive glands, as reflected in the principal component analysis presented in S2 Fig of the supporting information. Variations in these samples were caused by entirely different CoAs, therefore plotting at opposite directions in the PCA plot, whereas trends in all other organs could be explained by a similar set of CoAs. As digestive glands had the highest biomass, acyl-CoAs below the detection limit in stomach and hindgut samples could still be detected here. Interestingly, the second most abundant acyl-CoA in the digestive glands was propionyl-CoA. In contrast, succinyl-CoA was the second most abundant acyl-CoA in the stomachs and hindguts during both seasons, often followed by malonyl-CoA. While the latter two, like acetyl-CoA, are involved in numerous metabolic pathways, propionyl-CoA hints at the biosynthesis or degradation of odd-chain fatty acids or branched-chain amino acids [35,36]. Possible explanations for this phenomenon may include the activity of different metabolic pathways, variation of the microbiome across the three sections of the digestive system, or the prevalence of the krill-derived signal in the digestive gland due to the greater quantity of krill tissue compared to bacteria or food particles.

The composition of acyl-CoAs also varied between summer and autumn. For instance, 3-hydroxy-3-methylglutaryl-CoA was nearly absent in autumn samples, whereas it was found in significant amounts in all three organs in the summer samples. In contrast, U6-CoA, possibly nonenoyl-CoA, was relatively abundant in the digestive gland samples collected in autumn. Statistical analysis using the Kruskal-Wallis and Dunn’s tests indicated that pentanoyl-CoA was significantly more prevalent in autumn samples, while isopentanoyl-CoA was dominant in summer (data presented in S5 Table of the supporting information).

3-Hydroxy-3-methylglutaryl-CoA plays a role in various metabolic processes. It serves as an intermediate in the metabolism of the branched amino acids leucine, isoleucine and valine. Additionally, it is a crucial intermediate in isoprenoid biosynthesis, where it is converted to mevalonic acid by 3-hydroxy-3-methylglutaryl coenzyme A reductase. Crustaceans require this metabolic pathway, among others, to produce the juvenile hormone methyl farnesoate, which is involved in their reproductive processes [37]. Our data indicate that the metabolic pathways involving 3-hydroxy-3-methylglutaryl-CoA do not exhibit equal levels of activity throughout the year.

**Fig 2:**
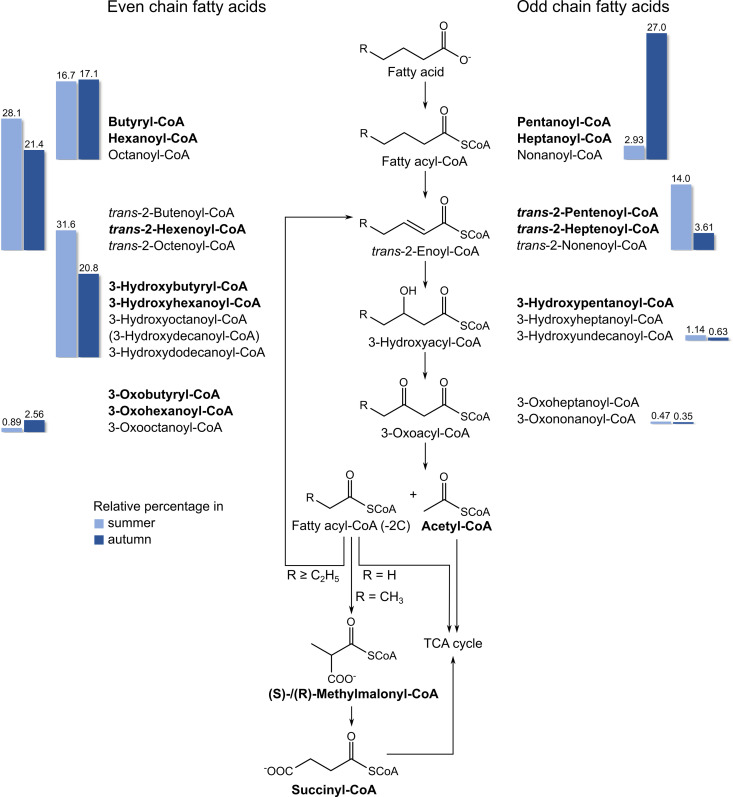
β-Oxidation of saturated even and odd chain fatty acids and detection of potentially related acyl-CoAs. Acetyl-CoA formed during β-oxidation of a fatty acid is channelled through the tricarboxylic acid (TCA) cycle. The remaining acyl-CoA, shortened by two carbon atoms, is processed by repeated β-oxidation. In the case of even-chain fatty acids, the final product is acetyl-CoA, which directly enters the TCA cycle. For odd-chain fatty acids, propionyl-CoA remains, which is converted to succinyl-CoA via (*S*)- and (*R*)-methylmalonyl-CoA, the latter two being inseparable with our analytical method, before it also enters the TCA cycle. The presence of bolded acyl-CoAs and acetyl-, succinyl- and methylmalonyl-CoA was confirmed by comparison with reference standards. The other acyl-CoAs listed in this figure were assigned through determination of the corresponding sum formulas by high-resolution mass spectrometry. In the case of isomeric acyl-CoAs with molecular formulas for which reference standards were unavailable, the retention sequence was used as an additional criterion. It was considered that acyl-CoA thioesters of unbranched alkanoic acids within a group of isomers have the highest retention times [25]. 3-Hydroxydecanoyl-CoA is shown in brackets, as it eluted earlier than heptanoyl-CoA and therefore did not correspond to the assumed retention time for this compound. The bar graphs depict the percentage of the different types of degradation products for even- and odd-chain fatty acids in summer (light blue) and autumn (dark blue) samples, with percentages indicated above the bars.

An important metabolic pathway to which a considerable portion of the acyl-CoAs could be assigned is the β-oxidation of saturated fatty acids; 29 detected acyl-CoAs, 16 verified by comparison with a reference standard, fall into this group (Fig 2).

Since lipids can comprise over 40% of the dry mass of krill, the presence of fatty acid degradation products in the form of acyl-CoAs is not surprising, although this has not been studied in krill [38]. Metabolites associated with the degradation of up to dodecanoic acid were identified. Overall, as expected, the relative proportions of acyl-CoAs associated with the β-oxidation of even-chain fatty acids were higher than those related to the β-oxidation of odd-chain fatty acid. An exception to this trend was the abundance of odd-chain fatty acyl-CoAs in autumn samples, mainly due to pentanoyl-CoA. In the majority of the stomach and hindgut samples from autumn, this acyl-CoA was the most abundant and the only acyl-CoA associated with β-oxidation. The composition of intact phospholipids from the same organs and during the same seasons also reflected a higher abundance of odd-chain fatty acids in autumn [15,39]. Therefore, acyl-CoAs may also serve as a valuable tracer for active metabolic pathways at the time of sampling.

### Amino acids

**Fig 3:**
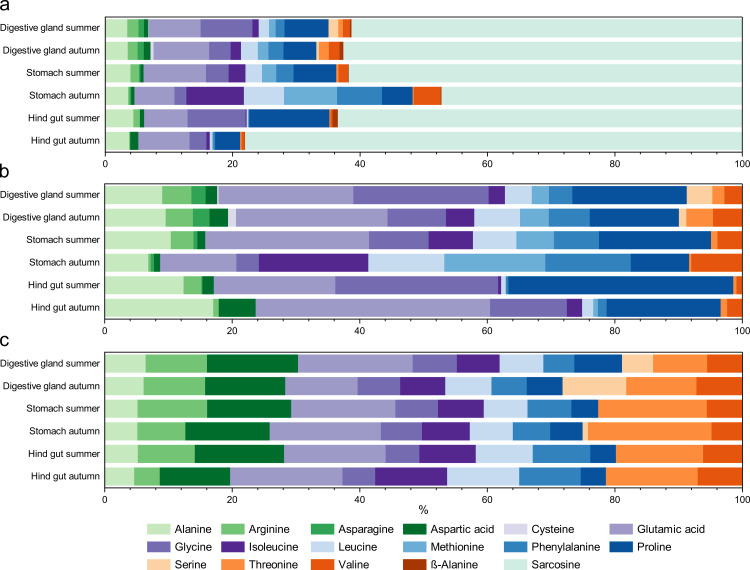
Relative distribution of amino acids between organs and seasons. The different diagrams show a. all free amino acids, b. only free proteinogenic amino acids and c. protein-bound amino acids.

During the amino acid analysis, 15 proteinogenic and two non-proteinogenic amino acids (β-alanine and sarcosine) were detected and quantified. Fig 3 shows the composition of the quantifiable free and protein-bound amino acids.

Interestingly, sarcosine had the highest relative amounts of all free amino acids at 47–78%. As sarcosine is not a proteinogenic amino acid, it does not occur in the protein-bound fraction. Sarcosine has previously been detected in krill [20] and, among other amino acids, in high proportions in muscle-free krill meal for fish aquaculture [40]. Sarcosine contents between 4 and 15% of the free amino acids were found in whole animals of three species of North Atlantic krill (*Meganyctiphanes norvegica, Thysanoessa inermis, T. raschii*) [41]. In another study, sarcosine was detected in the muscles *of M. norvegica and T. inermis*, but not in those of *E. superba* [42].

Sarcosine has also been identified in various other crustaceans. Significant amounts were found in trichloroacetic acid extracts from hepatopancreas and blood of the rock lobster (*Jasus lalandii*) [43]. The authors also mentioned that they had found sarcosine in the freshwater crab *Potamon* (*Potamonantes*) *perlatus/sydneyi*. Sarcosine was also found in considerable proportions (13–53% of the free amino acids) in the hepatopancreas of the snow crab (*Chionoecetes opilio*) [44]. During the analysis of 16 shrimp species (frozen animals) of different geographical origin, sarcosine was only detected in the two species analysed from the family *Pandilidae* (*Plesionika edwardsi, Pandalus montagui*) in amounts between 61 and 89 mg per 100 g sample [45]. Furthermore, sarcosine was detected in unspecified amounts in the metasomal muscle of freshwater amphipods from Lake Baikal [46] and in freshwater shrimps of the species *Macrobrachium rosenbergii* [47], whereby the sarcosine content of the latter was considerably higher in moulting animals than in the non-moulting control (C-stage).

Overall, the available findings seem to provide evidence that the occurrence of sarcosine in crustaceans is associated with their digestive tract and that sarcosine plays a role in the digestive process of these animals. However, the origin of sarcosine is unclear. One possibility is that it is produced by microorganisms in the crustacean’s digestive system. A recently published genome and transcriptome study has shown that the biosynthesis of sarcosine is absent in the genome of both prokaryotic and eukaryotic marine microorganisms, with the exception of Alphaproteobacteria, 55% of which had a synthesis pathway [48]. The authors concluded that the source of sarcosine in the ocean is relatively unknown. In contrast to biosynthesis, sarcosine degradation was far more common in both the prokaryotic and eukaryotic groups [48].

This suggests that, alternatively, the krill itself could produce the sarcosine detected in its digestive system. In fact, digestive juices of *Cancer pagarus*, a marine crustacean, contain emulsifiers identified as fatty acylsarcosyltaurines; these compounds along with a fatty acid and taurine contain sarcosine as a building block [49]. After injection of 1-^14^C acetate into *C. pagarus*, radioactive labeling was detected in the sarcosine contained in the emulsifiers. This finding proves that it was produced biosynthetically using acetate and is therefore of endogenous origin [50].

The high sarcosine level could furthermore be related to krill’s lipid metabolism, which is also reflected in the occurrence of acyl-CoAs involved in β-oxidation (see above). One of the most abundant lipids found in krill are phosphatidylcholines [15,51]. Its choline moiety may be converted to sarcosine through dimethylglycine as an intermediate, which is also detectable in our samples [48,52]. Consequently, high concentrations of choline in the form of phosphatidylcholine could elucidate why the metabolism is directed towards sarcosine production.

In addition to its involvement in digestive processes, sarcosine could also play a role as an osmolyte. In general, osmolytes are substances that fulfill cell-protective functions in organisms living under water stress, which may be related to salinity, temperature and pressure, among others, by increasing the osmotic pressure in the cytoplasm [53–55]. Three groups of low molecular weight organic osmolytes are known, namely polyols, amino acids (including sarcosine) and urea in combination with methylamines. Osmolytes occur in a wide variety of marine organisms [42,48,56,57]. A characteristic property of osmolytes is that they do not affect the structure of proteins, even at high concentrations, so that their functionality is not impaired, and that they can contribute to the stabilisation of proteins without being directly bound to them [58]. An elevated level of sarcosine may serve as an important protective factor by promoting the stabilisation of cold-adapted enzymes [59].

In a study examining human dietary supplementation with Antarctic krill oil, increased plasma levels of sarcosine were also found [52]. The authors attributed this to the fact that supplementation with choline directs human metabolism toward sarcosine production, possibly similar to its formation in the krill’s digestive system.

As the high sarcosine may mask the distribution patterns of the other amino acids, Fig 3b illustrates the composition while excluding the non-proteinogenic amino acids. Notably, the protein-bound amino acids were fairly evenly distributed across seasons and organs, with no significant differences, although this is not surprising given that the composition of protein amino acids is rather uniform even across different taxa [60]. At the same time, differences are observable in the free fraction, which is also reflected in the principal component analysis (S3 Fig of the supporting information). The most considerable differences were observed between the stomach samples of the two seasons, with significant variations found for alanine, arginine, glutamic acid, proline and valine (cf. S5 Table). The food entering the stomach, the first part of the digestive system, is not yet fully processed. Dietary-driven differences may be best seen in this organ, which is expected to reflect the freshest food signal.

Some amino acids, such as alanine, glutamic acid, glycine, and proline, were more abundant in the free form. In contrast, others, such as arginine, leucine, isoleucine, and threonine had a higher proportion in the protein-bound fraction when the stomach samples from autumn were excluded. These distributions and shifts are comparable with previously published data from whole krill [22,23,61], where greater variances in the content of free amino acids in whole krill between late summer and autumn were noted. Meanwhile, the distributions of the hydrolysed fractions were quite similar [22]. However, these differences were not as pronounced as those observed in the stomach samples. Therefore, while a seasonal trend might also be detected in whole krill samples, the most reliable indicators of recent food intake are found in the stomach.

The possibility that the high levels of propionyl-CoA in the digestive gland are an indication of increased metabolism of branched amino acids (see above) is not confirmed by the levels of isoleucine and valine found. Propionyl-CoA is therefore possibly an indicator of increased β-oxidation of odd-numbered fatty acids.

### B vitamins

Eight of the eleven B vitamins measured by our method were above the detection limit in the samples analysed. Of the cobalamins, which include cyano-, hydroxy-, methyl-, and adenosylcobalamin (AB_12_), only the latter was detected.

**Fig 4:**
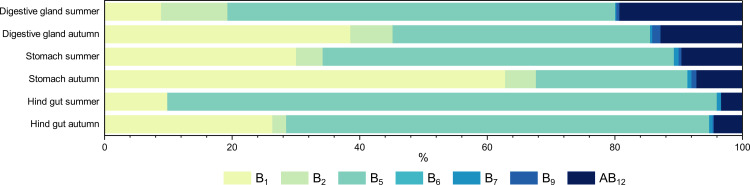
Relative distribution of B vitamins between organs and seasons. B_1_, thiamine; B_2_, riboflavin; B_5_, pantothenic acid; B_6_, pyridoxine; B_7_, biotin; B_9_, folic acid; AB_12_, adenosylcobalamine.

As illustrated in Fig 4, the composition of B vitamins varied across seasons and within organs. The observed trends, when comparing the relative amounts in the organs, remained largely consistent. However, the proportion of AB_12_ decreased from the digestive gland via the stomach to the hind gut. The two vitamins that had the greatest effect on the variation in seasonal patterns were B_1_ and B_5_. Summer samples consistently exhibited higher levels of B_5_, while B_1_ was more abundant in the autumn samples. Of these two vitamins, a statistical difference was only confirmed for B_1_, which had the highest impact on stomach samples taken in autumn (S5 Table and S4 Fig of the supporting information). Vitamin B_1_ is the coenzyme of numerous enzymes involved in acetyl-CoA synthesis, the tricarboxylic acid cycle, the pentose phosphate pathway, and in isoprenoid biosynthesis, among others [62]. Genes of twenty-one B_1_-requiring enzymes have been documented to be encoded in genomes of marine prokaryotes and eukaryotes, five of which (pyruvate dehydrogenase, acetolactate synthase, 1-deoxy-D-xylulose-5-phosphate synthase, transketolase, and α-ketoglutarate dehydrogenase) were present in all taxonomic groups examined (alphaproteobacteria, gammaproteobacteria, cyanobacteria, bacteroidetes, eukaryotes) [63]. It is reasonable that these enzymes also occur in the krill gut’s microbiome, implying the requirement for B_1_. Vitamin B_5_ constitutes an integral part of the coenzyme A molecule and, therefore, is essential for many metabolic pathways, such as fatty acid biosynthesis or energy production via the citric acid cycle [64]. Similar to other metabolic classes, the variations observed in vitamin B content may suggest either endogenous processes or reflect signals from recent food intake.

In a previous study, Kim et al. measured the monthly changes of B_1_, B_5_, and B_6_ in whole krill to determine their nutritional composition [22]. Contrary to our results, they documented high levels of B_6_ and only minor concentrations of B_5_. Additionally, they reported a decrease in B_1_ and B_6_ from summer to autumn, whereas our data suggest the opposite. These differences could be due to the fact that they measured the concentration in whole krill and not in isolated organs.

### Respiratory quinones

**Fig 5:**
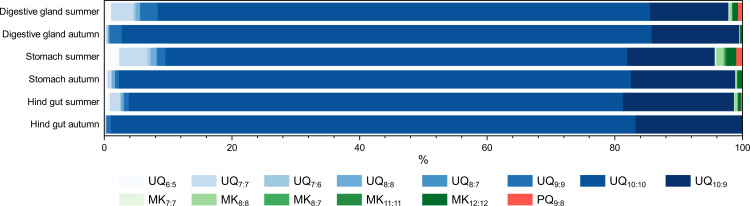
Relative distribution of respiratory quinones between organs and seasons. Types of ubiquinones are coloured in shades of blue, menaquinones in green and the plastoquinone in orange.

To our knowledge, the composition of respiratory quinones in krill, similar to acyl-CoAs, has not been previously investigated. As initial data for this class of biomolecules, we identified fourteen different respiratory quinones from three subclasses, namely eight ubiquinones (UQ), five menaquinones (MK) and one plastoquinone (PQ) (Fig 5). The most abundant respiratory quinone was UQ_10:10_, a 1,4-benzoquinone with 10 unsaturated isoprenyl subunits in the side chain, also referred to as coenzyme Q_10_. Like the essential omega-3 fatty acids obtained from krill, coenzyme Q_10_ plays an important role in human consumption as a dietary supplement [65,66]. Even though there are reports that UQ_9_ is the predominant respiratory quinone in Antarctic fish to achieve cold adaptation due to its lower crystallization temperature compared to UQ_10_, UQ_10_ is usually the predominant ubiquinone in other fishes and marine invertebrates [67–69].

Despite the generally similar distributions of respiratory quinones as reflected in the principal component analysis (S5 Fig of the supporting information), some seasonal variations can be observed. UQ_10:10_ and UQ_10:9_ predominated in the autumn samples, a pattern also noted in the summer samples, albeit with a higher proportion of other UQs, MKs, and PQ. Any variations in the composition of respiratory quinones may be related to dietary composition or adaptations of the digestive system due to changes in food intake, considering that there are several potential origins for the detected respiratory quinones [70].

As the name suggests, ubiquitous ubiquinones are found in almost all living organisms. They have also been detected in various bacterial isolates from the Antarctic marine environment [71–73]. UQ_8:8_ has been classified as the major respiratory quinone in a bacterium isolated from Antarctic krill [74]. Additionally, UQ_9:9_ was found in bacteria isolated from an Antarctic green alga, suggesting a potential dietary uptake of this respiratory quinone [75]. Menaquinones are mainly microbial respiratory quinones [70]. Several MKs, such as MK_7:7_ and MK_8:8_, have been described in Antarctic coastal marine environments [76,77]. Some bacteria can produce respiratory quinones with different degrees of unsaturation, providing a potential source of these kind of respiratory quinones, such as MK_8:7_ [78]. Plastoquinone 9 plays a significant role in photosynthesis, suggesting possibly phytoplankton uptake [79]. However, we detected only minimal amounts of PQ_9:8_, which contained one isoprenyl unit that was not unsaturated.

### Implications for krill environmental adaptation

Throughout the year, krill experience pronounced seasonal changes in their habitat, with low sea ice cover and almost constant daylight promoting extensive primary production, providing abundant food for krill during summer. In contrast, food becomes scarce due to shorter days and increased sea ice cover in winter. In this challenging habitat, krill maintain a substantial biomass which can sustain the entire Southern Ocean ecosystem. The ability of krill to thrive in such a challenging environment is attributed to their capacity to adapt to the extreme environmental conditions, including low temperatures and strong seasonal fluctuations [80].

Krill adapt to these seasonal fluctuations by regulating several physiological functions. During winter, krill enter a state of “energy-saving mode,” characterised by a halt in development and reproduction [81,82], an accumulation of lipids [38,83], and a reduction in overall metabolism [83,84], enabling them to survive the harsh winter conditions. In contrast, in spring, krill transition back to an “active mode,” with increased feeding activity, heightened metabolism, and active reproduction and development.

**Fig 6:**
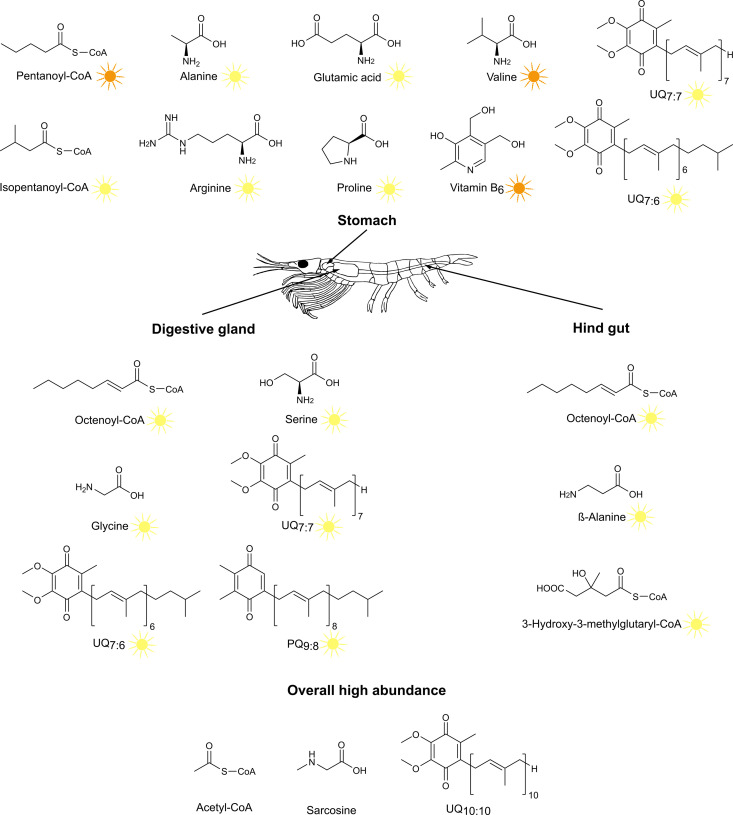
Summary of characteristic metabolites with significant differences between seasons for each organ. Metabolites marked with a yellow sun had a significantly higher abundance in summer samples, whereas metabolites with an orange sun where significantly more abundant in autumn samples.

In this study, we identified several metabolite classes that exhibited pronounced seasonal changes between summer and autumn samples (Fig 6). Among the acyl-CoAs, 3-hydroxy-3-methylglutaryl-CoA, a potential marker for reproductive processes [37], was detected in significant quantities throughout the entire digestive system in summer, while it was absent in autumn samples, aligning with the period of active reproduction in krill during that time of year. Vitamin B_5_, which is involved in fatty acid metabolism and energy production pathways [64], displayed heightened levels during krill’s active phase in summer, when overall metabolism is elevated.

In addition to its adaptation to seasonal fluctuations, krill is highly adapted to the cold waters of the Southern Ocean, where temperatures can drop to −2°C.

Interestingly, the amino acid sarcosine is found in high proportions (up to 78%) among all free amino acids, but shows no clear seasonal differences. It is conceivable that sarcosine is involved in the digestive processes in krill, but it could also play an important role in cold adaptation mechanisms [e.g., 85], which in turn reflects the adaptation of krill to the polar habitat. Seasonal fluctuations in water temperatures within the Southern Ocean are typically minimal, and krill must be prepared to encounter extremely low temperatures throughout the year. This may explain the absence of seasonal differences in sarcosine levels. In summary, our data reinforce the fundamental importance of krill’s adaptation to its extreme environment.

## Conclusion

In this study, classes of metabolic products that have rarely been investigated in krill or comparable species were analysed for the first time. These initial measurements provide a valuable basis for future evaluations and interpretations. The dataset presented in this study documents the enormous potential of important classes of metabolites that can be used as tracers to detect shifts in the composition of Antarctic krill due to changing environmental conditions or variations in its food supply. Further long-term datasets will additionally support and extend these new insights, allowing the development of useful new tools to detect small changes in the metabolome. Since *Euphausia superba* plays a central role in biogeochemical cycles and is also relevant for human consumption, early detection of changes in diet and, thus, in the environment can be crucial.

## Supporting information

S1 Table Full-scan orbitrap settings. Settings used for full-scan high-resolution mass spectrometry on the Orbitrap instrument for the detection of respiratory quinones.
(PDF)

S2 Table Targeted orbitrap settings. Settings for targeted mass spectrometry for the detection of respiratory quinones.
(PDF)

S3 Table Calibration ranges of B vitamins. (PDF)

S4 Table Detected coenzyme A thioesters. List of all peaks detected during coenzyme A measurements, including their exact masses, sum formulas and, where applicable, their identification.
(PDF)

S5 Table Results of the Kruskal-Wallis and Dunn’s tests where significances were observed. (PDF)

S1 Fig Relative distribution of all coenzyme A thioesters between organs and seasons. a. Including acetyl-CoA and b. excluding acetyl-CoA.
(PDF)

S2 Fig Principal component analysis of the percentage distribution of coenzyme A thioesters numbered according to S4 Table. Colours indicate the sampling months while shades and symbols represent the organ sampled from krill as indicated in the legend. Due to crowding on the right side, not all numbers are displayed.
(PDF)

S3 Fig Principal component analysis of the percentage distribution of free and protein-bound amino acids. Colours indicate the sampling months while shades and symbols represent the organ sampled from krill as indicated in the legend.
(PDF)

S4 Fig Principal component analysis of the percentage distribution of B vitamins. Colours indicate the sampling months while shades and symbols represent the organ sampled from krill as indicated in the legend.
(PDF)

S5 Fig Principal component analysis of the percentage distribution of respiratory quinones. Colours indicate the sampling months while shades and symbols represent the organ sampled from krill as indicated in the legend.
(PDF)
