# Supplementary material for: Variations of the metabolome in the digestive system of Antarctic krill, Euphausia superba, between summer and autumn
Source: PLoS One. 2025 Jul 10;20(7):e0327747. doi: 10.1371/journal.pone.0327747 (PMC12244748; doi:10.1371/journal.pone.0327747)
Supplement: S1 Table — Settings used for full-scan high-resolution mass spectrometry on the Orbitrap instrument for the detection of respiratory quinones. (PDF) [file pone.0327747.s001.pdf]

S1 Table. Full-scan orbitrap settings. Settings used for full-scan high-resolution mass spectrometry on the Orbitrap instrument for the detection of respiratory quinones.

Full scan settings	
Positive ion (V)	4100
Sheath gas (Arb)	40
Aux gas (Arb)	8
Sweep gas (Arb)	1
Ion transfer tube temperature (°C)	320
Vaporizer temperature (°C)	360
Orbitrap resolution	60.000
Scan range (*m/z*)	350-1250
RF lense (%)	60
AGC target	4E+05
Maximum injection time (ms)	50
MS^2 settings	
Intensity threshold	2.5E+04
Isolation window (*m/z*)	1.6
Collision energy mode	Stepped
HCD collision energies (%)	20, 35, 40
Orbitrap resolution	30.000
AGC target	1E+04
Maximum injection time (ms)	54
