# Supplementary material for: Variations of the metabolome in the digestive system of Antarctic krill, Euphausia superba, between summer and autumn
Source: PLoS One. 2025 Jul 10;20(7):e0327747. doi: 10.1371/journal.pone.0327747 (PMC12244748; doi:10.1371/journal.pone.0327747)
Supplement: S2 Table — Settings for targeted mass spectrometry for the detection of respiratory quinones. (PDF) [file pone.0327747.s002.pdf]

S2 Table. Targeted orbitrap settings. Settings for targeted mass spectrometry for the detection of respiratory quinones.

Targeted MS settings	
Positive ion (V)	4100
Sheath gas (Arb)	40
Aux gas (Arb)	8
Sweep gas (Arb)	1
Ion transfer tube temperature (°C)	320
Vaporizer temperature (°C)	360
MS^n levels (n)	2
Maximum number of multiplex ions	3
Isolation window (*m/z*)	1
HCD collision energies (%)	35 ± 5
Orbitrap resolution	60.000
Scan range (*m/z*)	140-1200
RF lense (%)	60
AGC target	5E+04
Maximum injection time (ms)	118
