# Supplementary material for: Variations of the metabolome in the digestive system of Antarctic krill, Euphausia superba, between summer and autumn
Source: PLoS One. 2025 Jul 10;20(7):e0327747. doi: 10.1371/journal.pone.0327747 (PMC12244748; doi:10.1371/journal.pone.0327747)
Supplement: S3 Table — (PDF) [file pone.0327747.s003.pdf]

S3 Table. Calibration ranges of B vitamins.

Vitamin	Concentration range (ng/mL)	Number of concentrations
B_1	0.5 - 550	29
B_2	0.2 - 550	31
B_3	1 - 275	27
B_5	0.7 - 1925	31
B_6	0.05 - 27.5	28
B_7	1.5 - 27.5	15
B_9	0.5 - 137.5	27
AB_{12}	3 - 550	26
