# Supplementary material for: Variations of the metabolome in the digestive system of Antarctic krill, Euphausia superba, between summer and autumn
Source: PLoS One. 2025 Jul 10;20(7):e0327747. doi: 10.1371/journal.pone.0327747 (PMC12244748; doi:10.1371/journal.pone.0327747)
Supplement: S4 Table — List of all peaks detected during coenzyme A measurements, including their exact masses, sum formulas and, where applicable, their identification. (PDF) [file pone.0327747.s004.pdf]

S4 Table. Detected coenzyme A thioesters. List of all peaks detected during coenzyme A measurements, including their exact masses, sum formulas and, where applicable, their identification.

Green CoAs were identified by comparison with reference standards. Red CoAs are proposals based on the sum formula, neglecting possible branches in most cases. CoAs marked with an * are those considered in Fig. 2.

RT	ESI+ $[M+H]^+$	Sum formula (-H)	Identification	Sum formula of acyl-rest	Nr.
6.68	840.1436	$C_{24}H_{40}N_7O_{18}P_3S$	3-Hydroxypropionyl-CoA	$C_3H_5O_2$	1
6.75	854.1231	$C_{24}H_{38}N_7O_{19}P_3S$	**Malonyl-CoA**	$C_3H_3O_3$	2
7.10	898.1491	$C_{26}H_{42}N_7O_{20}P_3S$	2-Hydroxyglutaryl-CoA	$C_5H_7O_4$	3
7.15	898.1491	$C_{26}H_{42}N_7O_{20}P_3S$	2-Hydroxyglutaryl-CoA	$C_5H_7O_4$	4
7.18	868.1385	$C_{25}H_{40}N_7O_{19}P_3S$	**Succinyl-CoA**	$C_4H_5O_3$	5
7.40	868.1385	$C_{25}H_{40}N_7O_{19}P_3S$	**Methylmalonyl-CoA**	$C_4H_5O_3$	6
7.60	882.1542	$C_{26}H_{42}N_7O_{19}P_3S$	**2-Methylsuccinyl-CoA**	$C_5H_7O_3$	7
7.70	912.1647	$C_{27}H_{44}N_7O_{20}P_3S$	**3-Hydroxy-3-methyglutaryl-CoA**	$C_6H_9O_4$	8
7.78	882.1542	$C_{26}H_{42}N_7O_{19}P_3S$	**3-Methylsuccinyl-CoA**	$C_5H_7O_3$	9
8.15	882.1544	$C_{26}H_{42}N_7O_{19}P_3S$	**Ethylmalonyl-CoA**	$C_5H_7O_3$	10
8.76	810.1331	$C_{23}H_{38}N_7O_{17}P_3S$	**Acetyl-CoA**	C_2H_3O	11
9.01	852.1436	$C_{25}H_{40}N_7O_{18}P_3S$	**Acetoacetyl-CoA***	$C_4H_5O_2$	12
9.39	854.1592	$C_{25}H_{42}N_7O_{18}P_3S$	**3-Hydroxybutyryl-CoA***	$C_4H_7O_2$	13
9.74	898.1855	$C_{27}H_{46}N_7O_{19}P_3S$	Dihydroxyhexanoyl-CoA	$C_6H_{11}O_3$	14
9.80	854.1593	$C_{25}H_{42}N_7O_{18}P_3S$	2-Hydroxybutyryl-CoA	$C_4H_7O_2$	15
10.38	868.1753	$C_{26}H_{44}N_7O_{18}P_3S$	3-Hydroxy-2-methylbutyryl-CoA	$C_5H_9O_2$	16
10.70	824.1494	$C_{24}H_{40}N_7O_{17}P_3S$	**Propionyl-CoA**	C_3H_5O	17
10.82	868.1753	$C_{26}H_{44}N_7O_{18}P_3S$	**3-Hydroxypentanoyl-CoA***	$C_5H_9O_2$	18
11.04	868.1753	$C_{26}H_{44}N_7O_{18}P_3S$	**3-Hydroxy-3-methylbutyryl-CoA**	$C_5H_9O_2$	19
11.27	836.1487	$C_{25}H_{40}N_7O_{17}P_3S$	Butenoyl-CoA*	C_4H_5O	20
11.76	882.1906	$C_{27}H_{46}N_7O_{18}P_3S$	Hydroxyhexanoyl-CoA	$C_6H_{11}O_2$	21
11.96	882.1906	$C_{27}H_{46}N_7O_{18}P_3S$	Hydroxyhexanoyl-CoA	$C_6H_{11}O_2$	22
12.04	880.1749	$C_{27}H_{44}N_7O_{18}P_3S$	Oxohexanoyl-CoA	$C_6H_9O_2$	23
12.11	836.1487	$C_{25}H_{40}N_7O_{17}P_3S$	**Crotonyl-CoA**	C_4H_5O	24
12.26	882.1906	$C_{27}H_{46}N_7O_{18}P_3S$	Hydroxyhexanoyl-CoA	$C_6H_{11}O_2$	25
12.59	880.1749	$C_{27}H_{44}N_7O_{18}P_3S$	Oxohexanoyl-CoA	$C_6H_9O_2$	26
12.73	838.1644	$C_{25}H_{42}N_7O_{17}P_3S$	**Isobutyryl-CoA**	C_4H_7O	27
12.84	880.1749	$C_{27}H_{44}N_7O_{18}P_3S$	**3-Oxohexanoyl-CoA***	$C_6H_9O_2$	28
13.00	838.1644	$C_{25}H_{42}N_7O_{17}P_3S$	**Butyryl-CoA***	C_4H_7O	29
13.17	904.1749	$C_{29}H_{44}N_7O_{18}P_3S$	Unknown	$C_8H_9O_2$	30
13.23	882.1906	$C_{27}H_{46}N_7O_{18}P_3S$	Hydroxyhexanoyl-CoA	$C_6H_{11}O_2$	31
13.30	894.1906	$C_{28}H_{46}N_7O_{18}P_3S$	Oxoheptanoyl-CoA	$C_7H_{11}O_2$	32
13.41	850.1644	$C_{26}H_{42}N_7O_{17}P_3S$	Pentenoyl-CoA	C_5H_7O	33
13.61	894.1906	$C_{28}H_{46}N_7O_{18}P_3S$	Oxoheptanoyl-CoA	$C_7H_{11}O_2$	34
13.66	850.1644	$C_{26}H_{42}N_7O_{17}P_3S$	Pentenoyl-CoA	C_5H_7O	35
13.77	882.1906	$C_{27}H_{46}N_7O_{18}P_3S$	**3-Hydroxyhexanoyl-CoA***	$C_6H_{11}O_2$	36
13.80	894.1906	$C_{28}H_{46}N_7O_{18}P_3S$	Oxoheptanoyl-CoA	$C_7H_{11}O_2$	37

13.88	896.2062	$C_{28}H_{48}N_7O_{18}P_3S$	Hydroxyheptanoyl-CoA	$C_7H_{13}O_2$	38
13.98	894.1906	$C_{28}H_{46}N_7O_{18}P_3S$	Oxoheptanoyl-CoA*	$C_7H_{11}O_2$	39
14.04	896.2062	$C_{28}H_{48}N_7O_{18}P_3S$	Hydroxyheptanoyl-CoA	$C_7H_{13}O_2$	40
14.28	896.2062	$C_{28}H_{48}N_7O_{18}P_3S$	Hydroxyheptanoyl-CoA	$C_7H_{13}O_2$	41
14.39	896.2062	$C_{28}H_{48}N_7O_{18}P_3S$	Hydroxyheptanoyl-CoA	$C_7H_{13}O_2$	42
14.46	850.1644	$C_{26}H_{42}N_7O_{17}P_3S$	**2-Methyl-2-butenoyl-CoA**	C_5H_7O	43
14.61	850.1644	$C_{26}H_{42}N_7O_{17}P_3S$	***trans*-Pentenoyl-CoA***	C_5H_7O	44
14.67	952.2324	$C_{31}H_{52}N_7O_{19}P_3S$	Decanedioyl-CoA	$C_{10}H_{17}O_3$	45
15.10	908.2062	$C_{29}H_{48}N_7O_{18}P_3S$	Oxooctanoyl-CoA	$C_8H_{13}O_2$	46
15.22	852.1802	$C_{26}H_{44}N_7O_{17}P_3S$	**2-Methylbutyryl-CoA**	C_5H_9O	47
15.43	852.1802	$C_{26}H_{44}N_7O_{17}P_3S$	**Isopentanoyl-CoA**	C_5H_9O	48
15.50	952.2324	$C_{31}H_{52}N_7O_{19}P_3S$	Decanedioyl-CoA	$C_{10}H_{17}O_3$	49
15.64	896.2062	$C_{28}H_{48}N_7O_{18}P_3S$	Hydroxyheptanoyl-CoA	$C_7H_{13}O_2$	50
15.68	908.2062	$C_{29}H_{48}N_7O_{18}P_3S$	Oxooctanoyl-CoA	$C_8H_{13}O_2$	51
15.96	896.2062	$C_{28}H_{48}N_7O_{18}P_3S$	Hydroxyheptanoyl-CoA*	$C_7H_{13}O_2$	52
15.96	852.1802	$C_{26}H_{44}N_7O_{17}P_3S$	**Pentanoyl-CoA***	C_5H_9O	53
16.19	910.2219	$C_{29}H_{50}N_7O_{18}P_3S$	Hydroxyoctanoyl-CoA	$C_8H_{15}O_2$	54
16.29	908.2062	$C_{29}H_{48}N_7O_{18}P_3S$	Oxooctanoyl-CoA	$C_8H_{13}O_2$	55
16.55	908.2062	$C_{29}H_{48}N_7O_{18}P_3S$	Oxooctanoyl-CoA	$C_8H_{13}O_2$	56
16.69	864.1800	$C_{27}H_{44}N_7O_{17}P_3S$	Hexenoyl-CoA	C_6H_9O	57
16.77	910.2219	$C_{29}H_{50}N_7O_{18}P_3S$	Hydroxyoctanoyl-CoA*	$C_8H_{15}O_2$	58
16.90	864.1800	$C_{27}H_{44}N_7O_{17}P_3S$	Hexenoyl-CoA	C_6H_9O	59
17.01	908.2062	$C_{29}H_{48}N_7O_{18}P_3S$	Oxooctanoyl-CoA	$C_8H_{13}O_2$	60
17.55	864.1800	$C_{27}H_{44}N_7O_{17}P_3S$	***trans*-Hexenoyl-CoA***	C_6H_9O	61
17.81	866.1957	$C_{27}H_{46}N_7O_{17}P_3S$	Hexanoyl-CoA	$C_6H_{11}O$	62
18.20	916.2113	$C_{31}H_{48}N_7O_{17}P_3S$	Perillyl-CoA	$C_{10}H_{13}O$	63
18.47	866.1957	$C_{27}H_{46}N_7O_{17}P_3S$	**Methylvaleryl-CoA**	$C_6H_{11}O$	64
18.86	866.1957	$C_{27}H_{46}N_7O_{17}P_3S$	Hexanoyl-CoA	$C_6H_{11}O$	65
19.41	866.1957	$C_{27}H_{46}N_7O_{17}P_3S$	**Hexanoyl-CoA***	$C_6H_{11}O$	66
19.66	878.1957	$C_{28}H_{46}N_7O_{17}P_3S$	**2-Methyl-2-hexenoyl-CoA**	$C_7H_{11}O$	67
20.11	878.1957	$C_{28}H_{46}N_7O_{17}P_3S$	***trans*-Heptenoyl-CoA***	$C_7H_{11}O$	68
20.59	880.2113	$C_{28}H_{48}N_7O_{17}P_3S$	Heptanoyl-CoA	$C_7H_{13}O$	69
21.30	938.2532	$C_{31}H_{54}N_7O_{18}P_3S$	Hydroxydecanoyl-CoA*	$C_{10}H_{19}O_2$	70
21.32	880.2113	$C_{28}H_{48}N_7O_{17}P_3S$	**2-Methylhexanoyl-CoA**	$C_7H_{13}O$	71
22.17	880.2113	$C_{28}H_{48}N_7O_{17}P_3S$	**5-Methylhexanoyl-CoA**	$C_7H_{13}O$	72
23.07	880.2113	$C_{28}H_{48}N_7O_{17}P_3S$	**Heptanoyl-CoA***	$C_7H_{13}O$	73
23.40	892.2113	$C_{29}H_{48}N_7O_{17}P_3S$	Octenoyl-CoA*	$C_8H_{13}O$	74
24.50	894.2270	$C_{29}H_{50}N_7O_{17}P_3S$	Octanoyl-CoA	$C_8H_{15}O$	75
24.65	906.2270	$C_{30}H_{50}N_7O_{18}P_3S$	Oxononanoyl-CoA*	$C_9H_{15}O_2$	76
24.80	906.2270	$C_{30}H_{50}N_7O_{17}P_3S$	Nonenoyl-CoA	$C_9H_{15}O$	77
25.22	906.2270	$C_{30}H_{50}N_7O_{17}P_3S$	Nonenoyl-CoA	$C_9H_{15}O$	78
25.50	894.2270	$C_{29}H_{50}N_7O_{17}P_3S$	Octanoyl-CoA	$C_8H_{15}O$	79
25.71	952.2688	$C_{32}H_{56}N_7O_{18}P_3S$	Hydroxyundecanoyl-CoA	$C_{11}H_{21}O_2$	80
25.75	906.2270	$C_{30}H_{50}N_7O_{17}P_3S$	Nonenoyl-CoA	$C_9H_{15}O$	81

26.08	962.2532	$C_{33}H_{54}N_7O_{18}P_3S$	Oxododecenoyl-CoA	$C_{12}H_{19}O_2$	82
26.10	908.2426	$C_{30}H_{52}N_7O_{17}P_3S$	Nonanoyl-CoA	$C_9H_{17}O$	83
26.40	966.2845	$C_{33}H_{58}N_7O_{18}P_3S$	Hydroxydodecanoyl-CoA	$C_{12}H_{23}O_2$	84
26.40	906.2270	$C_{30}H_{50}N_7O_{17}P_3S$	Nonenoyl-CoA	$C_9H_{15}O$	85
26.40	894.2270	$C_{29}H_{50}N_7O_{17}P_3S$	Octanoyl-CoA	$C_8H_{15}O$	86
26.60	894.2270	$C_{29}H_{50}N_7O_{17}P_3S$	Octanoyl-CoA	$C_8H_{15}O$	87
26.61	952.2688	$C_{32}H_{56}N_7O_{18}P_3S$	Hydroxyundecanoyl-CoA	$C_{11}H_{21}O_2$	88
26.67	908.2426	$C_{30}H_{52}N_7O_{17}P_3S$	Nonanoyl-CoA	$C_9H_{17}O$	89
26.80	918.2270	$C_{31}H_{50}N_7O_{17}P_3S$	Geranyl-CoA	$C_{10}H_{15}O$	90
26.80	906.2270	$C_{30}H_{50}N_7O_{17}P_3S$	Nonenoyl-CoA*	$C_9H_{15}O$	91
26.80	894.2270	$C_{29}H_{50}N_7O_{17}P_3S$	Octanoyl-CoA*	$C_8H_{15}O$	92
27.50	908.2426	$C_{30}H_{52}N_7O_{17}P_3S$	Nonanoyl-CoA	$C_9H_{17}O$	93
27.71	952.2688	$C_{32}H_{56}N_7O_{18}P_3S$	Hydroxyundecanoyl-CoA*	$C_{11}H_{21}O_2$	94
27.80	966.2845	$C_{33}H_{58}N_7O_{18}P_3S$	Hydroxydodecanoyl-CoA	$C_{12}H_{23}O_2$	95
28.74	966.2845	$C_{33}H_{58}N_7O_{18}P_3S$	Hydroxydodecanoyl-CoA*	$C_{12}H_{23}O_2$	96
28.80	908.2426	$C_{30}H_{52}N_7O_{17}P_3S$	Nonanoyl-CoA*	$C_9H_{17}O$	97
