# Supplementary material for: Variations of the metabolome in the digestive system of Antarctic krill, Euphausia superba, between summer and autumn
Source: PLoS One. 2025 Jul 10;20(7):e0327747. doi: 10.1371/journal.pone.0327747 (PMC12244748; doi:10.1371/journal.pone.0327747)
Supplement: S5 Table — (PDF) [file pone.0327747.s005.pdf]

S5 Table. Results of the Kruskal-Wallis and Dunn’s tests where significances were observed.

	Kruskal-Wallis Test						Dunn‘s Test									
Group	n	H	df	p	Eta^2	f	Group 1	Group 2	n1	n2	z	p	p.adj.	p.adj. signif.	r	
Free amino acids																
Alanine	83	30.286	5	1.30E-05	0.328	0.699	SA	SS	12	16	2.956	0.00312	0.04677	*	0.5586	
Arginine	83	35.355	5	1.28E-06	0.394	0.807	SA	SS	12	16	3.531	0.00041	0.00621	**	0.6673	
Glutamic acid	83	18.897	5	2.01E-03	0.180	0.469	SA	SS	12	16	3.299	0.00097	0.01454	*	0.6235	
Glycine	83	58.937	5	2.02E-11	0.700	1.529	DA	DS	12	16	3.610	0.00031	0.00459	**	0.6822	
Proline	83	22.376	5	4.44E-04	0.226	0.540	SA	SS	12	16	3.238	0.00121	0.01808	*	0.6119	
Serine	83	35.804	5	1.04E-06	0.400	0.817	DA	DS	12	16	3.723	0.00020	0.00296	**	0.7035	
Valine	83	55.931	5	8.40E-11	0.661	1.398	SA	SS	12	16	-3.172	0.00151	0.02270	*	-0.5995	
ß-Alanine	83	46.205	5	8.25E-09	0.535	1.073	HA	HS	11	16	3.516	0.00044	0.00658	**	0.6766	
B Vitamins																
B_1	65	45.079	5	1.40E-08	0.679	1.455	DA	DS	6	16	-3.908	0.00009	0.00139	**	-0.8333	
B_6	65	25.363	5	1.19E-04	0.345	0.726	SA	SS	5	16	-4.193	0.00003	0.00041	***	-0.9151	
Quinones																
$UQ_{7:7}$	63	35.292	5	1.32E-06	0.531	1.065	DA	DS	5	16	3.458	0.00054	0.00816	**	0.7546	
$UQ_{7:7}$	63	35.292	5	1.32E-06	0.531	1.065	SA	SS	5	16	3.025	0.00249	0.03732	*	0.6601	
$UQ_{7:6}$	63	32.476	5	4.78E-06	0.482	0.965	DA	DS	5	16	2.942	0.00326	0.04892	*	0.6420	
$UQ_{7:6}$	63	32.476	5	4.78E-06	0.482	0.965	SA	SS	5	16	3.375	0.00074	0.01109	*	0.7364	
$PQ_{9:8}$	63	23.067	5	3.28E-04	0.317	0.681	DA	DS	5	16	2.959	0.00308	0.04627	*	0.6457	
Coenzyme A thioesters																Nr. in S4 Table
2-Hydroxyglutaryl-CoA	64	18.198	5	2.71E-03	0.228	0.543	HA	HS	5	16	-3.634	0.00028	0.00418	**	-0.7930	4
3-Hydroxy-3-methylglutaryl-CoA	64	31.683	5	6.86E-06	0.460	0.923	HA	HS	5	16	3.754	0.00017	0.00261	**	0.8192	8#
Acetoacetyl-CoA	64	18.978	5	1.94E-03	0.241	0.563	HA	HS	5	16	3.030	0.00245	0.03669	*	0.6612	12
2-Hydroxybutyryl-CoA	64	51.434	5	7.05E-10	0.801	2.004	HA	HS	5	16	4.097	0.00004	0.00063	***	0.8940	15
Butenoyl-CoA	64	41.246	5	8.37E-08	0.625	1.291	HA	HS	5	16	3.966	0.00007	0.00110	**	0.8654	20
Hydroxyhexanoyl-CoA	64	54.836	5	1.41E-10	0.859	2.471	HA	HS	5	16	4.216	0.00002	0.00037	***	0.9201	21
Hydroxyhexanoyl-CoA	64	52.589	5	4.09E-10	0.820	2.138	HA	HS	5	16	4.139	0.00003	0.00052	***	0.9032	25
3-Oxohexanoyl-CoA	64	40.774	5	1.04E-07	0.617	1.269	HA	HS	5	16	3.644	0.00027	0.00403	**	0.7952	28
Unknown	64	43.305	5	3.21E-08	0.660	1.395	HA	HS	5	16	3.760	0.00017	0.00255	**	0.8204	30

Oxoheptanoyl-CoA	64	49.785	5	1.53E-09	0.772	1.841	HA	HS	5	16	4.022	0.00006	0.00087	***	0.8776	32
Pentenoyl-CoA	64	53.086	5	3.23E-10	0.829	2.202	HA	HS	5	16	4.157	0.00003	0.00048	***	0.9070	33
Oxoheptanoyl-CoA	64	44.138	5	2.17E-08	0.675	1.440	HA	HS	5	16	3.793	0.00015	0.00223	**	0.8278	34
Pentenoyl-CoA	64	48.166	5	3.28E-09	0.744	1.706	HA	HS	5	16	4.050	0.00005	0.00077	***	0.8838	35
3-Hydroxyhexanoyl-CoA	64	40.463	5	1.20E-07	0.611	1.254	HA	HS	5	16	3.632	0.00028	0.00422	**	0.7925	36
Oxoheptanoyl-CoA	64	44.256	5	2.05E-08	0.677	1.447	HA	HS	5	16	3.798	0.00015	0.00219	**	0.8288	37
Oxoheptanoyl-CoA	64	39.512	5	1.87E-07	0.595	1.212	HA	HS	5	16	3.533	0.00041	0.00617	**	0.7709	39
Hydroxyheptanoyl-CoA	64	31.311	5	8.13E-06	0.454	0.911	HA	HS	5	16	3.145	0.00166	0.02494	*	0.6862	42
trans-Pentenoyl-CoA	64	18.733	5	2.15E-03	0.237	0.557	DA	DS	6	16	3.231	0.00123	0.01848	*	0.6889	44
Decanedioyl-CoA	64	48.333	5	3.04E-09	0.747	1.719	HA	HS	5	16	3.907	0.00009	0.00140	**	0.8526	45
Oxooctanoyl-CoA	64	48.919	5	2.31E-09	0.757	1.766	HA	HS	5	16	3.991	0.00007	0.00099	***	0.8708	46
Isopentanoyl-CoA	64	34.915	5	1.56E-06	0.516	1.032	SA	SS	6	16	3.619	0.00030	0.00444	**	0.7716	48#
Decanedioyl-CoA	64	54.451	5	1.69E-10	0.853	2.405	HA	HS	5	16	4.203	0.00003	0.00040	***	0.9172	49
Hydroxyheptanoyl-CoA	64	39.592	5	1.81E-07	0.596	1.216	HA	HS	5	16	3.536	0.00041	0.00609	**	0.7717	50
Oxooctanoyl-CoA	64	54.867	5	1.39E-10	0.860	2.476	HA	HS	5	16	4.217	0.00002	0.00037	***	0.9202	51
Pentanoyl-CoA	64	46.088	5	8.71E-09	0.708	1.559	DA	DS	6	16	-3.973	0.00007	0.00107	**	-0.8470	53#
Pentanoyl-CoA	64	46.088	5	8.71E-09	0.708	1.559	SA	SS	6	16	-2.981	0.00287	0.04312	*	-0.6355	53#
Hydroxyoctanoyl-CoA	64	47.287	5	4.97E-09	0.729	1.640	HA	HS	5	16	3.060	0.00221	0.03317	*	0.6678	54
Hexenoyl-CoA	64	49.356	5	1.88E-09	0.765	1.803	HA	HS	5	16	3.477	0.00051	0.00759	**	0.7588	57
Hexenoyl-CoA	64	35.721	5	1.08E-06	0.530	1.061	HA	HS	5	16	3.280	0.00104	0.01560	*	0.7157	59
Oxooctanoyl-CoA	64	42.715	5	4.22E-08	0.650	1.364	HA	HS	5	16	3.904	0.00009	0.00142	**	0.8520	60
Hexanoyl-CoA	64	44.052	5	2.26E-08	0.673	1.436	HA	HS	5	16	4.016	0.00006	0.00089	***	0.8764	62
Methylvaleryl-CoA	64	49.040	5	2.18E-09	0.759	1.776	HA	HS	5	16	4.082	0.00004	0.00067	***	0.8908	64
2-Methyl-2-hexenoyl-CoA	64	27.363	5	4.85E-05	0.386	0.792	HA	HS	5	16	2.940	0.00328	0.04927	*	0.6415	67
trans-Heptenoyl-CoA	64	45.495	5	1.15E-08	0.698	1.521	HA	HS	5	16	3.962	0.00007	0.00112	**	0.8645	68
Heptanoyl-CoA	64	48.240	5	3.17E-09	0.746	1.712	HA	HS	5	16	3.903	0.00009	0.00142	**	0.8518	69
Hydroxydecanoyl-CoA	64	57.630	5	3.75E-11	0.907	3.131	HA	HS	5	16	4.266	0.00002	0.00030	***	0.9310	70
2-Methylhexanoyl-CoA	64	52.794	5	3.71E-10	0.824	2.164	HA	HS	5	16	4.083	0.00004	0.00067	***	0.8911	71
Octenoyl-CoA	64	42.317	5	5.08E-08	0.643	1.343	DA	DS	6	16	3.193	0.00141	0.02110	*	0.6808	74#
Octenoyl-CoA	64	42.317	5	5.08E-08	0.643	1.343	HA	HS	5	16	3.778	0.00016	0.00237	**	0.8244	74#
Octanoyl-CoA	64	54.206	5	1.90E-10	0.848	2.365	DA	DS	6	16	3.316	0.00091	0.01371	*	0.7069	75
Octanoyl-CoA	64	54.206	5	1.90E-10	0.848	2.365	HA	HS	5	16	4.195	0.00003	0.00041	***	0.9154	75
Nonenoyl-CoA	64	31.280	5	8.25E-06	0.453	0.910	HA	HS	5	16	3.143	0.00167	0.02507	*	0.6859	77

Octanoyl-CoA	64	54.966	5	1.33E-10	0.861	2.494	HA	HS	5	16	4.220	0.00002	0.00037	***	0.9209	79
Hydroxyundecanoyl-CoA	64	48.715	5	2.54E-09	0.754	1.749	HA	HS	5	16	3.983	0.00007	0.00102	**	0.8691	80
Nonenoyl-CoA	64	50.010	5	1.38E-09	0.776	1.861	HA	HS	5	16	3.058	0.00223	0.03339	*	0.6674	81
Oxododecenoic acid	64	26.498	5	7.14E-05	0.371	0.767	HA	HS	5	16	3.061	0.00221	0.03314	*	0.6679	82
Octanoyl-CoA	64	38.556	5	2.92E-07	0.579	1.172	HA	HS	5	16	3.641	0.00027	0.00407	**	0.7945	86
Hydroxyundecanoyl-CoA	64	54.596	5	1.58E-10	0.855	2.429	HA	HS	5	16	4.208	0.00003	0.00039	***	0.9182	88
Nonanoyl-CoA	64	43.847	5	2.49E-08	0.670	1.424	HA	HS	5	16	3.721	0.00020	0.00297	**	0.8121	89
Geranyl-CoA	64	41.103	5	8.94E-08	0.622	1.284	HA	HS	5	16	3.657	0.00026	0.00383	**	0.7980	90
Nonanoyl-CoA	64	51.640	5	6.39E-10	0.804	2.026	HA	HS	5	16	4.104	0.00004	0.00061	***	0.8957	93
Hydroxyundecanoyl-CoA	64	48.157	5	3.30E-09	0.744	1.705	HA	HS	5	16	3.157	0.00160	0.02394	*	0.6888	94
Hydroxydodecanoyl-CoA	64	27.376	5	4.82E-05	0.386	0.793	HA	HS	5	16	2.940	0.00328	0.04916	*	0.6417	95
Hydroxydodecanoyl-CoA	64	35.381	5	1.26E-06	0.524	1.049	HA	HS	5	16	3.343	0.00083	0.01244	*	0.7295	96

above 1% abundance in at least one sample

Relevant columns for the Kruskal-Wallis Test:

Group	Observed metabolite
n	number of observations
H	Chi² test statistic
df	degrees of freedom
p	significance (only if $p<0.05$, in which cases the null hypothesis (no difference observed) is rejected)
Eta²	measure for the effect size with values between 0 and 1 needed for the calculation of f
f	measure for the effect size with f > 0.1 = weak effect, f > 0.25 moderate effect an f > 0.4 = large effect [1]

Relevant columns for the Dunn’s Test:

Group 1 and Group 2	Compared tissue samples abbreviated with DA (digestive gland autumn), DS (digestive gland summer), SA (stomach autumn), SS (stomach summer), HA (hindgut autumn) and HS (hindgut summer)
n1	number of group 1 samples
n2	number of group 2 samples
z	standard test statistic
p	significance
p.adj.	p adjusted with Bonferroni correction
r	effect size. The interpretation values are: >0.1 (weak effect), >0.3 (moderate effect), >0.5 (large effect) [1]

References

1. Cohen J. A power primer. Psychol Bull. 1992; 112:155–9. doi: 10.1037//0033-2909.112.1.155 PMID: 19565683.
