# Supplementary material for: Variations of the metabolome in the digestive system of Antarctic krill, Euphausia superba, between summer and autumn
Source: PLoS One. 2025 Jul 10;20(7):e0327747. doi: 10.1371/journal.pone.0327747 (PMC12244748; doi:10.1371/journal.pone.0327747)
Supplement: S1 Fig — Including acetyl-CoA and b. excluding acetyl-CoA. (PDF) [file pone.0327747.s006.pdf]

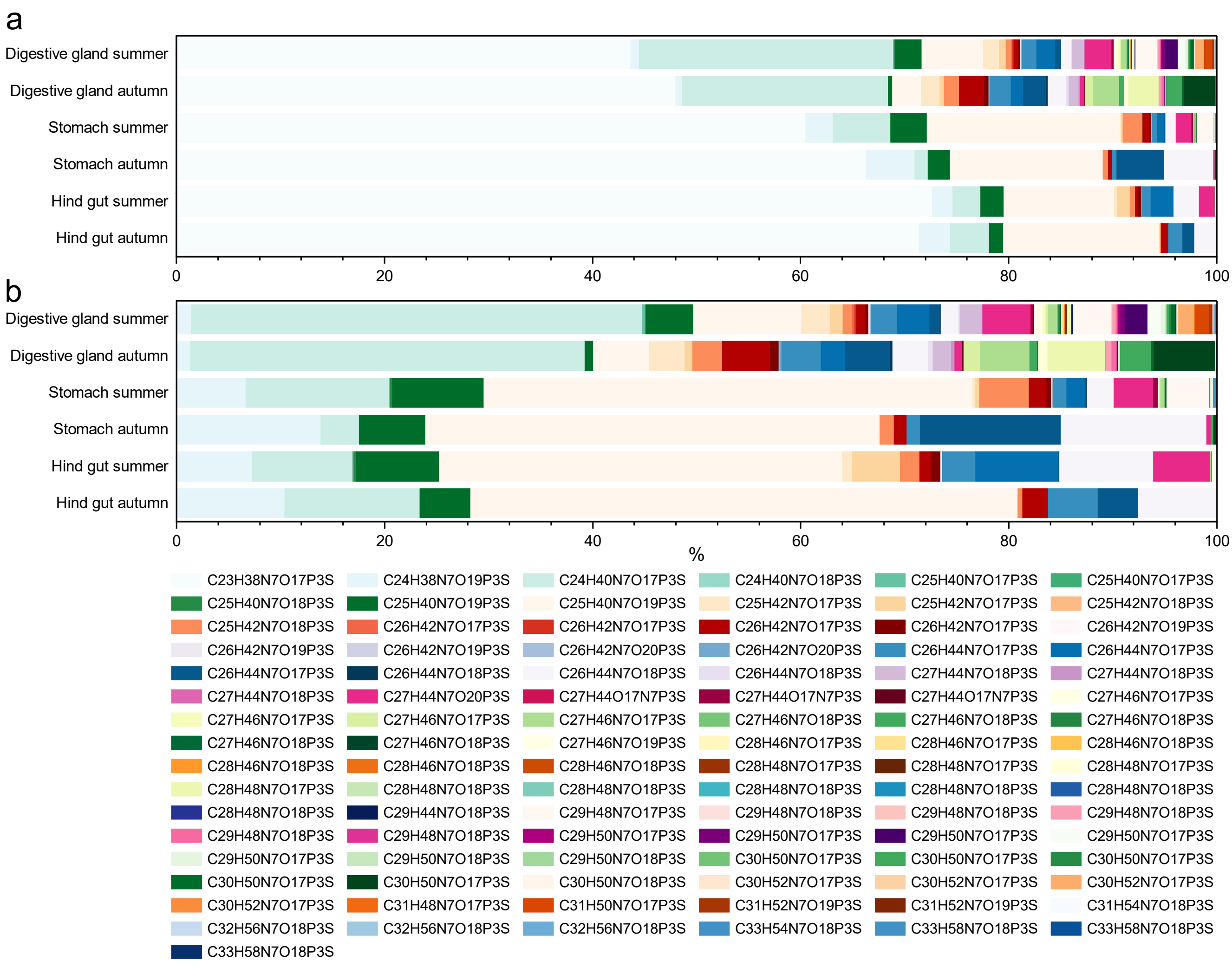

S1 Fig. Relative distribution of all coenzyme A thioesters between organs and seasons. a. Including acetyl-CoA and b. excluding acetyl-CoA.
