# Supplementary material for: Variations of the metabolome in the digestive system of Antarctic krill, Euphausia superba, between summer and autumn
Source: PLoS One. 2025 Jul 10;20(7):e0327747. doi: 10.1371/journal.pone.0327747 (PMC12244748; doi:10.1371/journal.pone.0327747)
Supplement: S2 Fig — Colours indicate the sampling months while shades and symbols represent the organ sampled from krill as indicated in the legend. Due to crowding on the right side, not all numbers are displayed. (PDF) [file pone.0327747.s007.pdf]

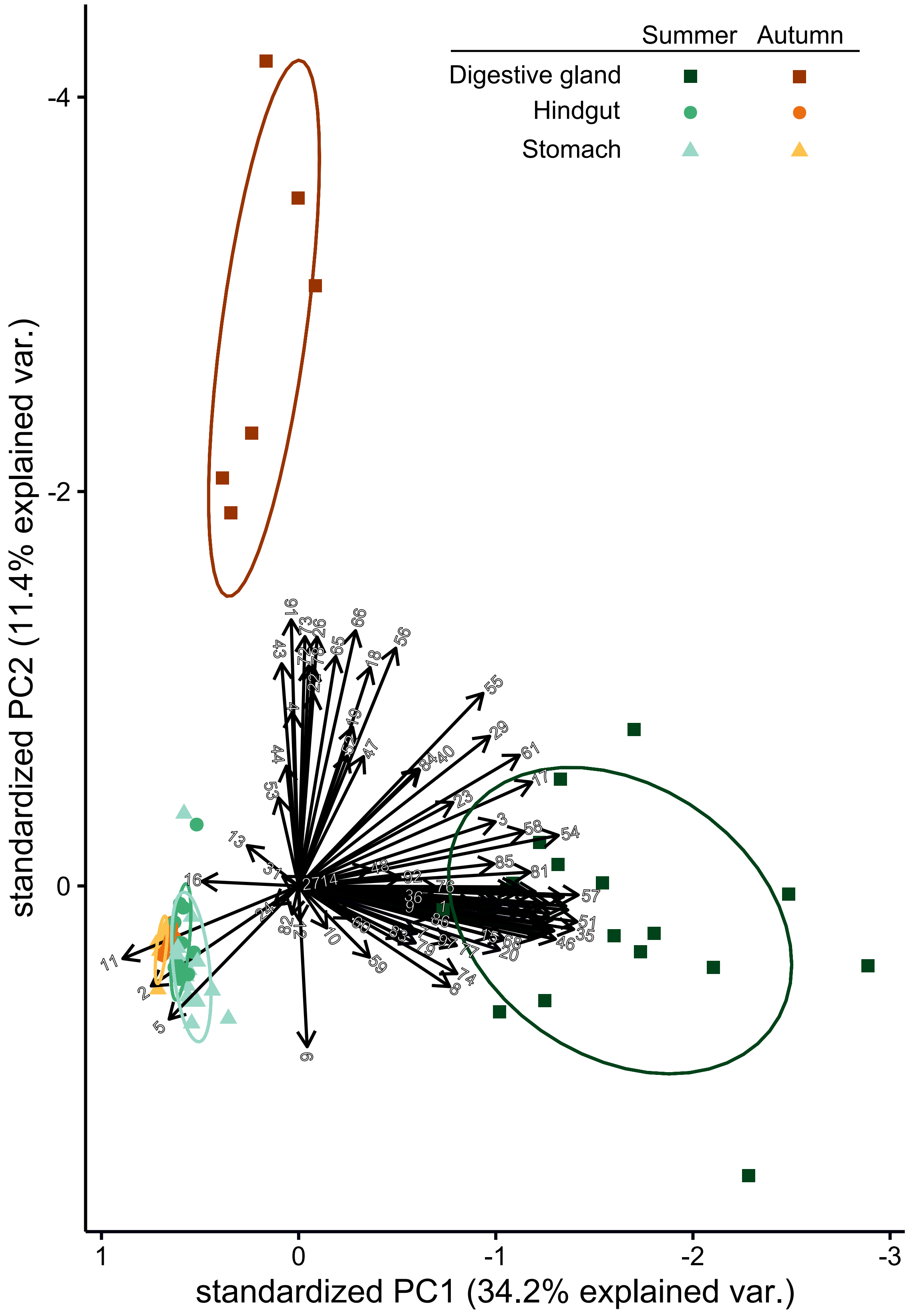


S2 Fig. Principal component analysis of the percentage distribution of coenzyme A thioesters numbered according to S4 Table. Colours indicate the sampling months while shades and symbols represent the organ sampled from krill as indicated in the legend. Due to crowding on the right side, not all numbers are displayed.
