# Supplementary material for: Variations of the metabolome in the digestive system of Antarctic krill, Euphausia superba, between summer and autumn
Source: PLoS One. 2025 Jul 10;20(7):e0327747. doi: 10.1371/journal.pone.0327747 (PMC12244748; doi:10.1371/journal.pone.0327747)
Supplement: S4 Fig — Colours indicate the sampling months while shades and symbols represent the organ sampled from krill as indicated in the legend. (PDF) [file pone.0327747.s009.pdf]

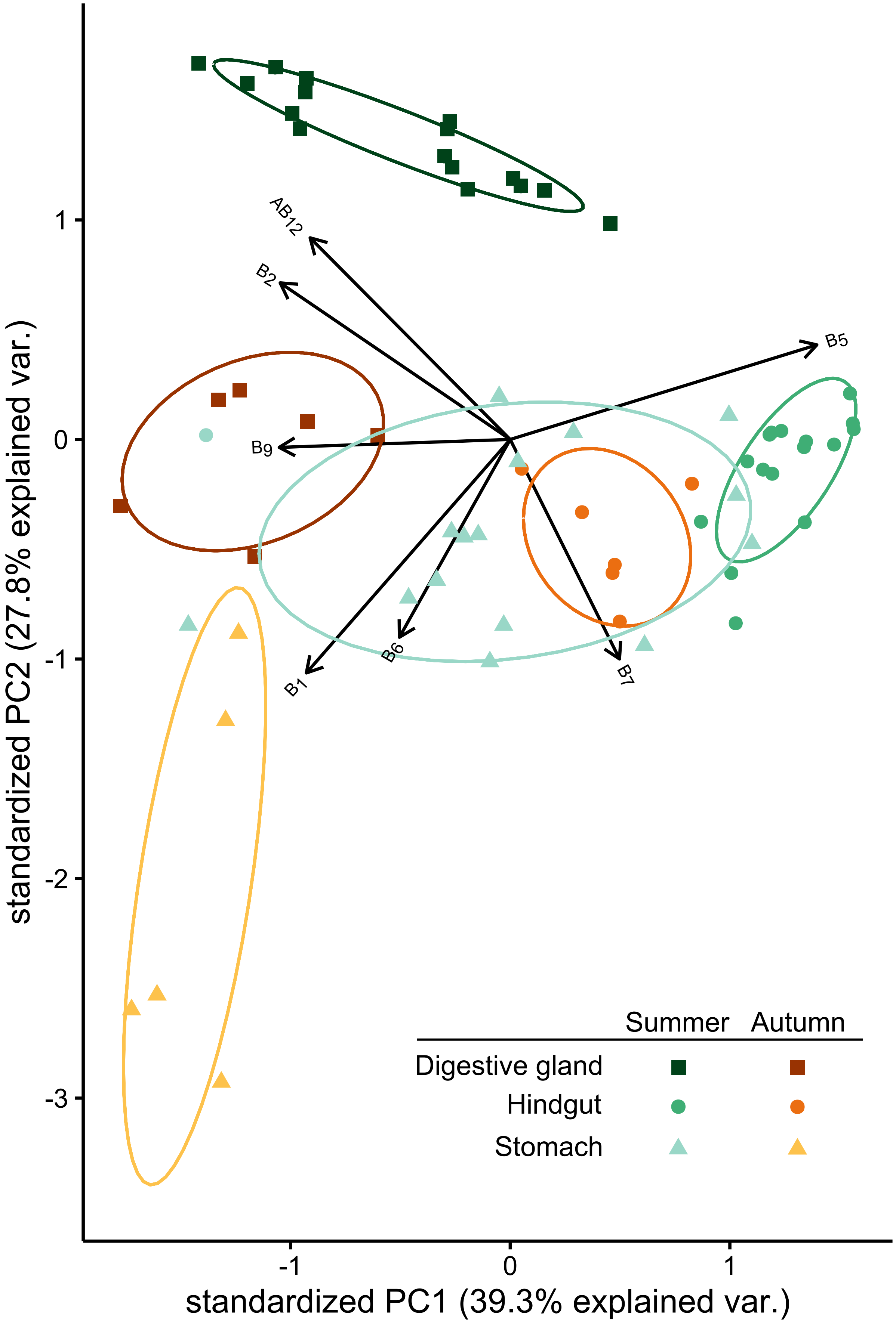

S4 Fig. Principal component analysis of the percentage distribution of B vitamins. Colours indicate the sampling months while shades and symbols represent the organ sampled from krill as indicated in the legend.
